# Jefferson Medical College

**Published:** 1844-02-24

**Authors:** H. T. Child


### CLINICAL LECTURES AND REPORTS.

### JEFFERSON MEDICAL COLLEGE.

*December* 13, 1843.

### CLINIC OF PROFESSOR MÜTTER.

(Reported by H. T. Child.)

Continued from last number.

#### CASE II.—TUMOUR OF THE TONGUE.

This patient, a female aged about 50 years, has perceived for the last six or eight weeks a gradual increase in the size of her tongue, particularly about the central and left half of the organ. The surface of the tumour is livid, with here and there an ulcerated spot, while its base is circumscribed. There is considerable lancinating pain in the affected part, and both speech and deglutition are materially interfered with. The general health of the patient is unimpaired; the glands in the neighbourhood are not enlarged; on the affected side there are several decayed teeth. There is nothing like cancer in the family of the patient, nor can she attribute the swelling to any manifest cause.

*Remarks.*—This case has rather a dangerous aspect, and would be pronounced, on a hasty examination, *cancer of the tongue*—but cancer generally begins in a different way, (either as a small and hard tubercle, a fungous growth, a figure, or an ulcer with a hardened base,) and is characterized by great pain, a florid color, enlargement of the neighboring lymphatic glands, and more or less disturbance of the general system.

I am therefore disposed to look upon this tumour as nothing more than the result of chronic inflammation of the tongue, characterized by a rather unusual deposite of coagulated lymph, and occasioned in all probability by the irritation of the carious teeth; we shall direct the removal of these teeth, and order her to take five grains of the Iodide of Potassium, three times a day—and also to bathe the parts with a solution of Potass. Iodid. ʒij.

Aqua destill. f℥iv. M. several times a day. Her bowels to be kept relaxed, and in order to promote absorption. A strictly vegetable diet was enjoined.

#### CASE III.—GELATINOUS POLYPUS OF THE NOSE.

The peculiarities of nasal polypus have been so often detailed, that I will not take up your time this morning with any additional remarks in relation to it.

This case is a well marked one; and I shall use in the removal of the tumour, the common polypus forceps. The tumour was then removed, the nostril sponged out, and the patient ordered to use several times a day, the following snuff:

℞. Cupri. Sulph. ℈j.

Pulv. Sanguinariæ Canad. ℈viij.

#### CASE IV.—ANEURISM BY ANASTOMOSIS.

This child, aged 4 months, presents the singular marks over each eye, which you perceive, and which have existed since birth. Their color is of the brightest red, but may be made to disappear to a certain extent by compression. Their is no pulsation, pain, or inconvenience at this time in or about these tumours, but they are *rapidly increasing* in size, and on this account it is proper to attempt their removal.

*Remarks.*—These tumours belong to the class of affections included under the denomination, *nævus maternus*, or *mother's mark*, and which are attributed by the ignorant to impressions made upon the mother's mind, and transmitted by her to the infant. But as this term includes other and very dissimilar affections, surgeons have distinguished the affection under consideration by various appellations, derived from the structure of the tumour itself. Thus it is called *erectile tumour*, *congenital varicose tumour*, *aneurism by anastomosis*, *hematon us*, *telangiectasis*, *arteriectasis*, *aneurisma verum cylindroideum*, *tissue splenoide*, *&c. &c.* The tumor is composed of a tissue of arterial and venous capillary vessels, held together by cellular substance, and may be developed in every organ and tissue of the body, varying in *size*, *shape*, *colour*, *consistence* and *depth*, is *elastic* to the touch, and often *pulsates*, or yields a *thrilling sensation*, in consequence of each tumour being supplied (usually) by an artery of some size.

If the tumour is seated on or in an important organ of large size or grows rapidly, it is often a troublesome affection, and inasmuch as it may, even when small, soon become serious from enlargement, it is best to resort to some operation for its relief, as soon as the child is old enough to bear it. The danger to be apprehended from its neglect, is ulceration, and the consequent hemorrhage which has proved fatal in some cases. Various methods of treatment have been introduced into practice; I will barely mention them, and then show you the operation which I generally prefer,—that in which the tumour is transfixed by one or two needles passed through its base, and then strangulated by a strong ligature.

The various operations are

1st. Compression. 2d. Ligature of the main arterial trunk. 3d. Encircling the tumour by incisions. 4th. The seton. 5th. Breaking up of the cells. 6th. Puncture, followed by the caustic probe—that is a silver probe dipped in strong nitric acid, thus forming an impure nitrate of silver. 7th. Puncture and injection of some stimulating fluid. 8th. Vaccination. 9th. Caustic potash. 10th. Nitric acid. 11th. Tartarized antimony et potassa. 12th. The actual cautery. 13th. Incisions under the skin. 14th. Acupuncture. 15th. Darning. 16th. Ligature of the whole mass. 17th. Excision. 18th. Tattooing.

